# [Retracted] TIPE-2 suppresses growth and aggressiveness of hepatocellular carcinoma cells through downregulation of the phosphoinositide 3-kinase/AKT signaling pathway

**DOI:** 10.3892/mmr.2026.13788

**Published:** 2026-01-05

**Authors:** Lin Wang, Chen Chen, Shuzhi Feng, Jianli Tian

Mol Med Rep 17: 7017–7026, 2018; DOI: 10.3892/mmr.2018.8789

Following the publication of the above paper, it was drawn to the Editor's attention by a concerned reader that certain of the immunofluorescence data shown in Fig. 1F, cell viability assay data in Fig. 2F, the ‘Control’ data for the tissue images relating to apoptotic experiments in Fig. 5B and the ‘CHOP/TIPE-2’ histological data in Fig. 5E were strikingly similar to data in articles written by different authors at different research institutes that had either already been published previously in other journals, or which were submitted for publication at around the same time (in the interim, one of those articles has been retracted). In addition, western blot data featured in Fig. 2 were rather similar to data appearing in Fig. 3, suggesting that the same data may have been included in these figures to show the results from purportedly differently performed experiments.

The Editorial Office were able to draw the same conclusions as the reader based upon an independent analysis of the contentious data in question. Therefore, owing to the fact that some of these data had already been published prior to its submission to *Molecular Medicine Reports*, the Editor has decided that this paper should be retracted from the Journal. The authors were asked for an explanation to account for these concerns, but the Editorial Office did not receive a reply. The Editor apologizes to the readership for any inconvenience caused.

